# Alkaline Amino Acids for Salt Reduction in Surimi: A Review

**DOI:** 10.3390/foods14142545

**Published:** 2025-07-21

**Authors:** Tong Shi, Guxia Wang, Yu Xie, Wengang Jin, Xin Wang, Mengzhe Li, Yuanxiu Liu, Li Yuan

**Affiliations:** 1 School of Food and Biological Engineering, Jiangsu University, No. 301, Xuefu Road, Zhenjiang 212013, China; 1000005720@ujs.edu.cn (T.S.); 18193389815@163.com (G.W.); 15707962778@163.com (Y.X.); wangxinbjt1015@163.com (X.W.); lmz19980805@163.com (M.L.); xiu0421@foxmail.com (Y.L.); 2 Bio-Resources Key Laboratory of Shaanxi Province, School of Biological Science and Engineering, Shaanxi University of Technology, Hanzhong 723001, China

**Keywords:** alkaline amino acids, surimi products, salt reduction, quality preservation, myofibrillar protein

## Abstract

Surimi products are popular due to their high protein and low fat content. However, traditional processing methods rely on high concentrations of salt (2–3%) to maintain texture and stability, contributing to excessive sodium intake. As global health trends advance, developing green and low-salt technologies while maintaining product quality has become a research focus. Alkaline amino acids regulate protein conformation and intermolecular interactions through charge shielding, hydrogen bond topology, metal chelation, and hydration to compensate for the defects of solubility, gelation, and emulsification stability in the low-salt system. This article systematically reviews the mechanisms and applications of alkaline amino acids in reducing salt and maintaining quality in surimi. Research indicates that alkaline amino acids regulate the conformational changes of myofibrillar proteins through electrostatic shielding, hydrogen bond topology construction, and metal chelation, significantly improving gel strength, water retention, and emulsion stability in low-salt systems, with the results comparable to those in high-salt systems. Future research should optimize addition strategies using computational simulations technologies and establish a quality and safety evaluation system to promote industrial application. This review provides a theoretical basis for the green processing and functional enhancement of surimi products, which could have significant academic and industrial value.

## 1. Introduction

**Figure 1:**
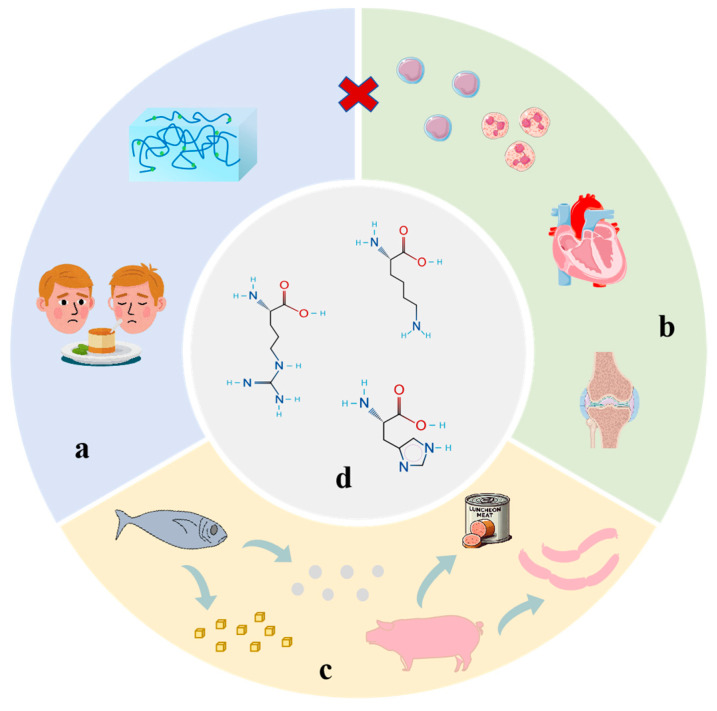
(**a**) The effects of reduced salt on surimi products. (**b**) The harm of high salt to human health. (**c**) Preparing gel products under high-salt conditions. (**d**) Molecular formulas of arginine, lysine, and histidine.

Surimi products, which primarily consist of myofibrillar protein, are traditional foods such as fish balls and crab sticks that are made through processes like chopping, mixing, and heating to form a gel. Thanks to their high protein content, low fat, and convenience, these products have become an integral part of the world’s culinary culture [1,2]. However, the WHO recommends < 5 g/day sodium intake. Furthermore, the 2% to 3% salt content in traditional surimi processing can increase the risk of hypertension and cardiovascular diseases [3]; excessive use of phosphates can lead to calcium and phosphorus metabolic disorders [4]. Reducing the salt content often results in decreased gel strength and texture quality in surimi [5,6]. Therefore, developing scientifically effective methods to reduce salt while maintaining quality in surimi products has become an urgent scientific challenge in the aquatic processing industry (Figure 1a–c).

Currently, salt reduction strategies often focus on ion replacements (such as KCl) or the addition of hydrophilic colloids, but these methods can easily lead to metallic odors or texture degradation [7,8]. Recent studies have shown that alkaline amino acids (Figure 1d), such as arginine, lysine, and histidine, can effectively compensate for the functional deficiencies of NaCl in low-salt systems due to their amphoteric properties and physiological activities [9,10]. For example, L-Lys has a highly electronegative ε-amino group, making it strongly basic [11]; L-Arg’s guanidinium group is positively charged under all conditions, playing a crucial role in protein interactions [12]; and L-His can provide proton donors and acceptors, offering antioxidant capabilities [13]. Furthermore, Guo et al. found that alkaline amino acids can enhance the solubility of myosin in low-NaCl solutions [13], improve the emulsification activity index/emulsification stability index, and reduce the emulsification index and droplet size [14,15], thereby enhancing the quality of meat products.

Despite the promising prospects, current research faces three major limitations: (1) mechanism analysis is often limited to a single fish species, such as silver carp, overlooking the impact of ω-3 fatty acid oxidation on amino acid function in high-fat fish like mackerel [16,17]; (2) the dose–response relationship is unclear, and excessive addition may lead to the loss of elasticity or flavor changes [18,19]; (3) the evaluation system relies heavily on subjective sensory analysis, lacking precise characterization of micro-networks and antibacterial properties [19].

Beyond functional limitations, scaling alkaline amino acid applications necessitates evaluating toxicological and regulatory hurdles. For instance, histidine may provoke histamine-related allergies in sensitive populations. Moreover, metabolic consequences, such as altered nitrogen metabolism in lysinuric protein intolerance (LPI) patients, also warrant caution [20]. In addition, there are regulatory differences between alkaline acids. The Food and Drug Administration (FDA) has only explicitly given Generally Recognized As Safe (GRAS) certification for arginine, and there is no uniform dosage standard for lysine and histidine, which complicates industrial applications.

This paper systematically reviews the molecular mechanism, functional improvement effect, and technical challenges of alkaline amino acids in salt reduction and quality preservation of surimi and puts forward future research directions by combining interdisciplinary strategies (such as molecular dynamics simulation and computational simulations technologies), aiming to provide innovative ideas and theoretical support for the development of healthy surimi products.

## 2. Structure and Functional Characteristics of Myofibrillar Protein in Surimi Products

### 2.1. The Key Role of Myofibrillar Protein in the Formation of Surimi Gel

The quality of surimi products fundamentally depends on the dynamic process of extraction, conformational regulation, and thermal-induced gelation of myofibrillar proteins (MPs). As the primary functional component of the surimi matrix (accounting for 60–75% of total protein), myofibrillar proteins are primarily composed of myosin heavy chains (MHC), actin, tropomyosin, and troponin. Among these, the actomyosin complex formed by myosin and actin constitutes over 80% of the total protein content in the system [21].

As a typical rod-shaped macromolecule (molecular weight approximately 520 kDa), the functional domains of myosin exhibit significant heterogeneity: the N-terminal contains ATPase active sites and actin-binding domains that are regulated by conformational changes through ATP hydrolysis, and the α-helix tail region self-assembles [22] through hydrophobic interactions. Low-temperature plasma treatment significantly increases the hydrophobicity and solubility of silver carp myosin, which is closely related to the reduction in α-helix content leading to molecular unfolding [23,24].

During heat processing, conformational changes in myosin play a key role in constructing the gel network. At temperatures between 40 and 60 °C, the head domain of myosin unfolds, exposing thiol groups, which form primary aggregates through intermolecular disulfide bonds (S-S). When the temperature exceeds 60 °C, the tail α-helix unfolds, leading to the formation of fibrous supramolecular structures [25,26]. Small-Angle X-ray Scattering (SAXS) confirmed that after 70 °C heat treatment under 0.5 M NaCl conditions, silver carp exhibited characteristic scattering peaks (q = 0.2–0.5 nm^−1^) corresponding to 15–25 nm nanopore structures, which are significantly positively correlated with the gel’s water-holding capacity (WHC > 90%) [24,27].

It is worth noting that the NaCl concentration significantly influences the gel properties by modulating the protein solvation layer. When the ionic strength increases from 0.1 M to 0.6 M, the solubility of myosin in the surimi increases by 2.3 times and the surface zeta potential decreases from −12.5 mV to −5.8 mV, which facilitates the formation of a uniform and dense network structure (the elastic modulus G′ increases from 125 Pa to 680 Pa) [28,29]. However, exceeding the critical concentration (0.8 M) leads to excessive swelling, disrupting the hydrogen-bond-mediated β-sheet assembly [30,31].

### 2.2. Mechanism of Action of NaCl on the Functional Characteristics of Surimi

#### 2.2.1. Solubility Regulation: Electrostatic Shielding and Myofilament Depolymerization

In a neutral, salt-free environment, myosin fibrils form stable aggregates due to surface charge repulsion, making them difficult to dissolve. When the NaCl concentration reaches 0.4–0.6 M, the electrostatic shielding effect induced by ionic strength can neutralize over 90% of the surface charges (with potential approaching zero), facilitating the dissociation of myosin heavy chains (MHC) from the thick filaments [32]. Synchrotron X-ray diffraction reveals that 0.5 M NaCl can expand the d-spacing in salmon myosin fibrils, indicating significant relaxation of the filament structure. At this point, the solubility of myosin can exceed 85%, providing a sufficient amount of monomer [33] for subsequent thermal gelation.

#### 2.2.2. Gel Strengthening: Conformational Transformation and Crosslinking Coordination

At a NaCl concentration of 2–3% (*w*/*v*), the rheological properties and thermal denaturation responsiveness of protein solutions are significantly enhanced. NaCl enhances the gel network through two mechanisms: first, by conformational regulation, where 0.3–0.6 M NaCl promotes the conversion of α-helices in the tail region of myosin to β-folds, thereby increasing the intermolecular hydrogen bond density [34,35]; second, by cross-linking promotion, where the exposure of hydrophobic groups (ANS fluorescence intensity increases by 2.1 times) and the release of active thiol groups (from 5.2 to 8.7 μmol/g) jointly drive a non-covalent–covalent cross-linking cascade reaction. Dynamic rheology indicates that the storage modulus (G′) of the 0.5 M NaCl system at a final temperature of 90 °C can reach 2250 Pa, which is 18 times higher than in the absence of salt [36,37].

#### 2.2.3. Optimization of Emulsification Stability: Interfacial Adsorption Kinetics

Myofibrillar proteins exhibit good interfacial activity, which is enhanced in the presence of NaCl, facilitating their adsorption on the oil–water interface and the formation of a stable emulsion film [38]. The adsorption capacity of myosin on the oil–water interface is positively correlated with the NaCl concentration. Wang et al. found that 0.4 M NaCl can increase the interfacial dilatational modulus of salmon myosin from 12.3 mN/m to 28.7 mN/m and reduce the average particle size of the emulsion to 1.2 μm [39]. NaCl also enhances protein flexibility, improving the adsorption rate and density of myofibrillar proteins at the interface, effectively preventing the aggregation of fat particles or emulsion breakdown and enhancing the uniform distribution of fats in surimi products [40,41]. Confocal laser scanning microscopy (CLSM) further confirmed that salt treatment improves the continuity of the protein membrane, effectively inhibiting fat globule aggregation (aggregation rate < 5%/24 h) [42].

### 2.3. Scientific Challenges and Solutions for a Low-Salt Surimi System

#### 2.3.1. Drastic Reduction in Protein Solubility and Extraction Efficiency

The primary issues caused by insufficient NaCl are the decrease in solubility and the reduction in protein extraction efficiency. Salt increases the ionic strength, promoting the depolymerization and dissolution of myosin and other myofibrillar proteins into the aqueous phase. Zhang [43] noted that when the NaCl concentration drops below 0.2 M, the solubility of myosin sharply decreases to below 30%. At this point, myofibril bundles (with a diameter greater than 200 nm) are difficult to dissociate, resulting in insufficient protein network density (porosity > 60%). Ultrasonic assistance (20 kHz, 300 W) combined with 0.1 M KCl can partially replace NaCl, restoring the protein extraction rate of cod roe to 75% [44,45,46]. Simultaneously, KCl partially replacing NaCl can effectively inhibit the formation of histamine, putrescine, and cadaverine in smoked perch [47]. However, the use of KCl is mainly limited by its bitter and astringent taste, especially at high substitution levels (>50% NaCl substitution), which significantly weaken the intensity of flavor and produce undesirable bitterness [48].

#### 2.3.2. Defects of Gel Network and Texture Degradation

Loose gel networks and poor texture are common defects in low-salt surimi. During gel formation, the tails of myosin proteins undergo heat-induced aggregation, forming a three-dimensional network structure. Low-salt (<0.15 M) conditions cause the gel’s G′ to drop to 150–300 Pa, resulting in a 25–40% [49] loss in water-holding capacity (WHC). Small-angle neutron scattering (SANS) reveals that the pore size distribution of the network broadens (10–100 nm) and the β-sheet order decreases, leading to a loose gel network, reduced elasticity, and an inability to effectively retain water, which results in decreased water-holding capacity and juice exudation (syneresis) [50]. Introducing 0.5% hydroxypropyl methylcellulose (HPMC) can restore the WHC to 85% [51,52] through hydrogen bonding compensation. Additionally, enzymatic cross-linking, induced by transglutaminase (TGase, 10 U/g) to form ε-(γ-glutamyl)lysine covalent bonds, can increase G′ by 2.8 times [53,54].

#### 2.3.3. Emulsion Instability and Phase Separation Risk

The reduction in emulsion stability is due to the salt concentration affecting the ability of myofibrillar proteins to form a stable emulsion film at the water–oil interface. Myofibrillar proteins can adsorb onto the surface of fat globules in the presence of an appropriate amount of NaCl, forming an orderly and dense protein film that stabilizes the emulsion system. Under low-salt conditions (<0.3 M), the flocculation rate constant of the tuna emulsion increases by 4.7 times and the adsorption amount (Γ) of interfacial proteins decreases to 1.2 mg/m^2^, leading to fat aggregation, floating, or precipitation, which affects the uniformity of the emulsion and the consistency of the texture [13]. Adding a 0.1% tea polyphenol–chitosan complex can enhance the elasticity of the interfacial film (E’ increases by 56%) and inhibit fat floating (oil precipitation rate < 3%) [55,56].

#### 2.3.4. Microbial Safety and Shelf-Life Bottleneck

The reduced shelf life and increased microbial risks pose significant challenges for the market promotion of low-salt products. Salt helps reduce water activity and inhibit bacterial growth, particularly against Gram-positive bacteria and some spoilage bacteria. In a low-salt environment, surimi is more prone to microbial spoilage and sensory degradation during storage [45,57]. Reducing the salt concentration from 2% to 0.5% can increase water activity, which in turn accelerates the growth rate of psychrotrophic bacteria, such as Pseudomonas [45]. Therefore, modern preservation techniques, including high-pressure treatment, the addition of antimicrobial peptides, and cold-chain assurance, are necessary to maintain product hygiene, safety, and shelf life. For example, high-pressure processing (HPP, 400 MPa/5 min) can effectively inhibit the growth of microorganisms like *Escherichia coli*, *Pseudomonas*, and *coagulase-positive Staphylococcus aureus*, thereby extending the refrigerated shelf life [57,58]. Additionally, nisin (200 IU/g), a natural antimicrobial peptide, can effectively inhibit the growth of spoilage bacteria. When used in conjunction with HPP, it can further extend the shelf life [7].

## 3. Physicochemical Properties of Alkaline Amino Acids and Their Interaction Mechanism with Myofibrillar Protein

Alkaline amino acids, including arginine, lysine, and histidine, exhibit a dual regulatory effect in surimi salt reduction processing characterized by their unique charged groups and molecular flexibility. This effect involves charge compensation, structural stabilization, and functional enhancement. This section systematically elucidates the mechanisms of these effects using molecular interaction characterization techniques, such as isothermal titration calorimetry (ITC), molecular docking, and atomic force microscopy (AFM) force spectroscopy.

### 3.1. Arginine

#### 3.1.1. Topological Construction of Hydrogen Bond Network

**Figure 2:**
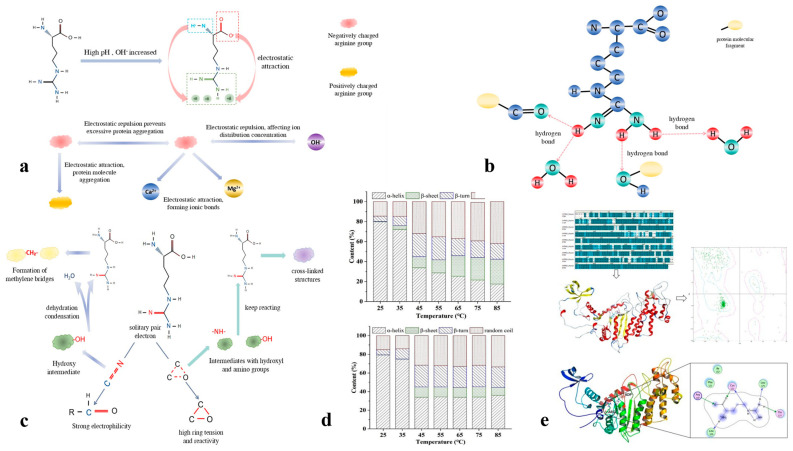
Effects of arginine on the structure, solubility, and gelling properties of surimi myofibrillar proteins. (**a**) The electrostatic interaction mechanism between Arginine and ions at high pH levels; (**b**) Effect of guanidine on gel stability; (**c**) Mechanism of crosslinking between amino acid and aldehyde group; (**d**) Homologous modeling and molecular docking of myg heavy chain protein [63]; (**e**). Effect of guanidinium on the secondary structures of myosin during heating (pH 7.0) [66].

The guanidinium group of arginine (pKa ≈ 12.5) carries a strong positive charge under neutral conditions and forms strong interactions with acidic residues in myosin (such as Glu/Asp) through double hydrogen bonds (binding constant K ≈ 1.2 × 10^4^ M^−1^) [59]. Circular dichroism (CD) shows that 0.5% Arg can increase the α-helix content of myosin from 31% to 38%, and the thermal denaturation temperature increases by 4.2 °C [60]. Wang et al. found that the addition of Arg enhances the stability of myosin’s α-helix and β-structure [61]. Cryo-electron microscopy (Cryo-EM) confirmed that Arg forms a regular network [62] with a spacing of 12.8 ± 0.3 nm by hydrogen bonding between adjacent myosin heads. The hydrogen bonds formed between the guanidinium group of arginine and water molecules or other molecules enhance the gel consistency of surimi (Figure 2a), improving its texture and elasticity [63,64,65].

#### 3.1.2. Π-Cation Interaction

The π system of the guanidinium group in arginine forms a strong π-cation interaction with aromatic amino acids (such as Tyr and Trp), which has a binding energy of −8.2 kcal/mol (as shown by molecular dynamics simulations) [67]. This type of interaction is common in protein structures, and arginine (Arg) tends to form such interactions more readily than lysine (Lys). Among aromatic amino acids, tryptophan (Trp) has the strongest binding ability [68,69]. Additionally, the strength of the cation-π interaction is significantly influenced by molecular hydration (Figure 2b); weak hydration facilitates the formation of strong interactions in water [70,71].

#### 3.1.3. Charge Shielding and Colloid Stability Regulation

In a low-salt system (0.1 M NaCl), 0.3% Arg can significantly increase the zeta potential of myosin from −25.3 mV to −8.7 mV; this change optimizes capillary water capture, thereby increasing the water-holding capacity (WHC) from 75% to 90% [72], effectively inhibiting its aggregation [73,74]. This effect may be due to Arg interacting with the aromatic and charged side chains on the myosin surface, which forms a stable interface that reduces the electrostatic repulsion between proteins. Additionally, the self-assembly properties of Arg, such as the formation of head–tail hydrogen bonds, may occupy protein surface space, thereby inhibiting protein–protein interactions and preventing aggregation. These mechanisms work together to stabilize myosin structures at low concentrations, enhancing their solubility and stability (Figure 2c).

### 3.2. Lysine

#### 3.2.1. Dynamic Protonation Regulation

**Figure 3:**
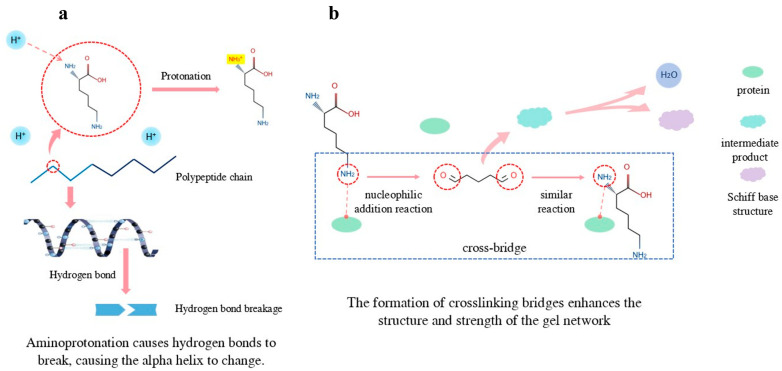
Effects of lysine on the structure and gelling properties of surimi myofibrillar proteins. (**a**) Effect of lysine protonation on α-helical structure; (**b**) Schematic diagram of crosslinking bridge formation in arginine gel network.

The ε-amino group of lysine (pKa ≈ 10.5) exhibits controlled protonation within the 6.0–8.0 pH range, which anchors the negatively charged regions of actin, such as the Asp-25 cluster, through electrostatic attraction [75]. At low pH, the amino group of lysine becomes protonated (Figure 3a), enhancing its interaction with other negatively charged amino acid residues. At high pH, the amino group of lysine deprotonates, increasing its hydrophilicity and altering the protein’s solubility and gel behavior. Isothermal titration calorimetry (ITC) measurements show that the enthalpy change (ΔH) for the binding of Lys to actin is −15.8 kJ/mol, indicating a strongly exothermic interaction [76].

#### 3.2.2. Maillard-Assisted Crosslinking

During heat treatment, the ε-amino group of Lys reacts with reducing sugars (such as glucose) through a Maillard reaction, forming ε-N-(carboxymethyl)lysine (CML). This process begins with the dehydration condensation reaction between the amino group of lysine and the carbonyl group of reducing sugars, forming Schiff bases (Figure 3b). These Schiff bases then transform into Amadori compounds, ultimately leading to the formation of CML [14,55] through a series of intermediate products. The formation of CML is influenced by heating temperature, sugar type (lactose > glucose > sucrose) [72], and heating time. The generated CML can cross-link with myosin thiol groups, increasing the gel’s storage modulus G′ by 2.3 times [55].

#### 3.2.3. Directed Assembly of Hydrate Layer

The amino group of lysine is highly hydrophilic, capable of binding up to 12–15 water molecules through its hydrophilic side chains (as shown by quasi-elastic neutron scattering data), forming a hydration layer that can be as thick as 1.8 nm. NMR analysis indicates that this hydration layer converts free water into bound water through a network of hydrogen bonds, reducing the loss rate of low-salt surimi during cooking by 62% [66,70]. This property of lysine enhances the water retention and water-holding capacity of gel products, maintaining freshness and texture, especially by preventing drying during processing [77,78,79].

### 3.3. Histidine

#### Proton Buffering Effect of Imidazole Ring

**Figure 4:**
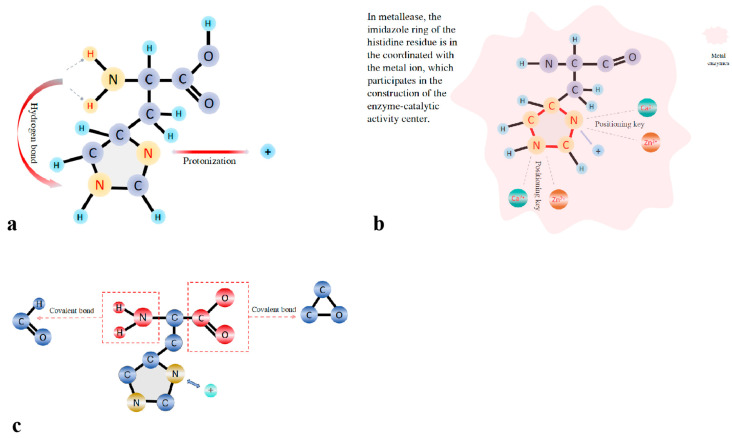
Effects of histidine on the structure and gelling properties of surimi myofibrillar proteins. (**a**) Histidine protonation and its effect on structure; (**b**) Coordination of histidine imidazole ring with metal ion in metalloproteinase; (**c**) The mechanism of condensation between histidine and carbonyl groups.

The histidine imidazole ring (pKa ≈ 6.0) exhibits bidirectional buffering capabilities within the surimi processing pH window (6.5–7.5) [80]. In an acidic microenvironment, the imidazole ring accepts protons to form His^+^, neutralizing negative charges on the protein surface (potential correction Δζ ≈ +7.3 mV). In a basic micro-environment, it releases protons to restore neutrality (Figure 4a), preventing excessive aggregation [22,81]. This characteristic helps regulate pH in surimi products, improving protein solubility and gel properties [50].3.3.2. His–Zn^2+^: Metal-Coordination Enhancement Mechanism in Low-Salt Surimi Gel.

The imidazole ring of histidine (pKa ≈ 6.0) forms a tetrahedral complex with metal ions (Zn^2+^, Ca^2+^) through its N3 atom, with a binding constant logK of 5.8 [50]. This complex acts as a ‘molecular rivet,’ bridging adjacent myosin molecules and promoting cross-linking, thereby enhancing the stability of the gel network [59]. This mechanism (Figure 4b,c) allows the gel strength of low-salt surimi (0.3% NaCl) to reach 98% of that of traditional formulations (2.5% NaCl). Atomic force microscopy (AFM) force spectroscopy shows that the His-Zn^2+^ complex increases the interprotein binding force from 0.6 nN to 2.1 nN. X-ray absorption fine structure (EXAFS) confirms that Zn^2+^ coordinates with two His imidazole rings and two Glu carboxyl groups, forming a stable four-coordinate structure [81,82].

## 4. The Effect of Alkaline Amino Acids on the Functional Characteristics of Surimi Products

### 4.1. Improve the Solubility of Myofibrillar Protein

Lysine, arginine, and histidine carry positive charges at neutral pH, which allows them to form salt bonds or salt bridges with carboxyl and anionic groups on myosin molecules. This increases the net charge and electrostatic repulsion of the protein, effectively moving it away from the isoelectric point and enhancing its solubility [80,83]. For example, adding arginine or histidine to a surimi protein solution increases the pH from 6.82 to 8.74 and 7.24, respectively, significantly deviating from the isoelectric point of myosin. This helps inhibit heat-induced aggregation and reduce protein particle size (Table 1) [84,85,86,87].

Lysine or arginine molecules can interact with exposed aromatic residues in the hydrophobic regions of proteins, inhibiting protein chain aggregation. Guo et al. found that adding 5 mmol/L L-His or L-Lys at a NaCl concentration of 0.1–0.6 mol/L results in more thiol and aromatic groups being exposed on myosin, while the α-helix content decreases, indicating protein chain unfolding, enhancing interaction with water molecules and ultimately improving solubility [13]. Additionally, Chen et al. proposed that at an extremely low ionic strength of 1 mmol/L KCl, 5 mmol/L L-His can disrupt α-helices, inhibiting myosin fiber aggregation and significantly increasing solubility [81]. When Lys or Arg is added, the hydration ability and surface hydrophobicity of myosin increase, while the hydrodynamic diameter decreases, significantly reducing aggregation and thus greatly improving solubility [84,88,89]. Studies also show that adding high concentrations of lysine or arginine under very low salt conditions can restore myosin solubility to levels comparable to high-salt controls (Table 1) [80].

### 4.2. Improve Emulsification Performance and Interfacial Stability

Alkaline amino acids can alter the secondary structure of proteins, exposing hydrophobic regions and enhancing flexibility, which facilitates the adsorption and arrangement of protein molecules at the oil–water interface. Experiments show that after Arg/Lys treatment, the β-sheet content in myofibrils increases, while the random coil content decreases, leading to a more ‘uncoiled’ protein conformation [90]. This conformational adjustment enhances surface activity. During stirring, treated proteins are more likely to form a dense and ordered protein film at the oil–water interface. Adding Arg/Lys significantly increases the absolute zeta potential (ζ-potential) (Table 1) of proteins in the emulsion, resulting in stronger electrostatic repulsion and hydrophobic interactions at the oil droplet interface, effectively inhibiting droplet aggregation and phase separation [32,62].

Lysine and arginine can accumulate at the oil–water interface, interacting with the surrounding water and lipid molecules through hydrogen or ionic bonds, stabilizing the membrane structure. Furthermore, replacing sodium salts with alkaline amino acids and adding specific components (e.g., eggs and soybean oil to surimi) followed by strong stirring can enhance emulsifying activity and stability [13,91]. Moreover, the amino acid composition of this emulsion aligns with the FAO/WHO recommended model, where the lysine content is twice the recommended level, suggesting lysine may indirectly enhance emulsifying properties by improving the amino acid balance.

### 4.3. Enhance the Thermal Gel and Water Retention Ability

During heat processing, lysine and arginine promote the cross-linking and polymerization of proteins, forming a more compact network structure. These amino acids can act as cross-linking sites (for example, lysine is prone to ε-γ-glutamyl cross-linking reactions) or enhance protein chain interactions through hydrogen bonding [92,93]. Additionally, they strengthen hydrophobic interactions, enhancing the interplay between protein fibers. The synergistic effect of Lys and microwave heating results in a denser network structure in low-salt surimi gels. SEM observations revealed a dense gel framework and that Lys treatment enhanced the formation of hydrogen bonds and disulfide bonds in the gel [94,95].

The addition of alkaline amino acids often enhances the thermal stability of proteins, increasing the enthalpy change required for gel formation. This results in more water being retained within the protein network during heating, thereby increasing the water-holding capacity [96,97]. For example, Wang [62] found that adding 15–20 mmol/L of Lys or Arg to a low-salt system significantly increased the water-holding capacity (WHC) and gel strength of myofibrillar proteins to levels comparable to those under high-salt conditions (Table 1). This treatment increases the proportion of ‘bound water’ in the gel, indicating that more water is trapped within the dense protein network [98].

After adding alkaline amino acids, the texture parameters of surimi gel, including elasticity, adhesion, and chewiness, were significantly enhanced. When 0.1% arginine is combined with different levels of oxidized caffeic acid (OCF), the gel strength and WHC of low-salt surimi gel can be significantly improved, especially when combined with 0.5% OCF (LC-A-O) (Table 1) [8]. Compared to the low-salt control, LC-A-O shows a significant improvement in water distribution and a marked increase in β-sheet structure content (*p* < 0.05). Additionally, the network structure of LC-A-O is more compact and uniform, possibly due to the formation of new complexes between OCF and surimi proteins leading to stronger and more intramolecular cross-linking. Therefore, the combined treatment of Arg and OCF shows potential for improving the characteristics of low-salt surimi gel. However, at present, this work is mainly concentrated at the laboratory scale, and its industrial feasibility needs to be further verified. We suggest that future studies should verify process reproducibility through pilot-scale experiments and monitor the dynamic changes of β-folding to assess industrial feasibility.

**Table 1:** Effects of Lys, Arg, and His on myofibrillar particle size, ζ potential, solubility, water retention capacity (WHC), and gel strength in low-salt systems.

Item	NaCl Concentration	L-Lysine	L-Arginine	L-Histidine	Material	Ref.
Particle Size	0.1 M	0.1% addition reduced droplet size from 2.5 to 1.8 μm, with uniform distribution	0.1% addition reduced droplet size from 2.5 to 1.9 μm, inhibiting aggregation	1 g L^−1^ addition reduced droplet size from 2.7 to 1.6 μm, improving emulsion stability	Myosin-stabilized emulsion	[99]
Zeta Potential	0.1 M	Increased from −25.3 to −8.7 mV	Increased from −12.5 to −5.8 mV	Absolute value decreased, suppressing aggregation	Shrimp/fish/chicken surimi	[100]
Solubility	0.1–0.6 M	>30% increase (from ~60 to >90%)	Rose from <30% to 85%	Increased by 22.3%, inhibiting aggregation	Porcine/fish/chicken myosin	[101]
Water- Holding Capacity (WHC)	1%	Rose from 60% to 85%	Rose from 62% to 89%	Decreased from 66.7% to 40.3% (requires optimization)	Shrimp surimi/beef paste	[100]
Gel Strength	1%	Enhanced by 378.83% (vs. low-salt control ≈ 440 g·mm)	Reached 1676.56 g·mm	Increased from 0.10 N to 0.22 N (≈120% increase)	Shrimp surimi/beef paste	[100]

## 5. Limitations and Future Prospects of Current Research

### 5.1. Limitations of the Study

#### 5.1.1. Lack of Singularity of Research Objects and Universality of Mechanism

Most existing studies focus on single categories such as fish balls and fish cakes, but the effects of different fish raw materials (such as high-fat and low-fat fish) and processing techniques (such as chopping intensity and heat treatment conditions) on the mechanisms of alkaline amino acids have not been systematically elucidated. Due to significant differences in muscle fiber protein composition and fat content among different fish species (such as salmon and mackerel), there may be heterogeneity in the ionic binding capacity, antioxidant efficacy, and antibacterial effects of alkaline amino acids, which limits the general applicability of the current conclusions.

#### 5.1.2. Standardization of Dosage and Risk of Side Effects

It is worth noting that JECFA (1973, 1974, 1975) listed L-Lys, L-Arg, and L-His as having an Acceptable Daily Intake (ADI). The European Food Safety Authority (2019) also confirmed that these amino acids are safe as food ingredients at a dose of ≤3%. However, the current research lacks a unified standard for the threshold of adding alkaline amino acids, with a wide range of dosages and an unclear interaction mechanism with the surimi matrix (such as pH and water activity) [102,103,104]. There are significant variations in the amount and ratio of alkaline amino acids added, and there is a lack of clear optimization guidance. Over-addition can lead to poor texture (such as reduced elasticity) or flavor shifts (such as residual metallic taste), while low doses struggle to balance the reduction in sodium content with the need for quality preservation. There is an urgent need to establish an optimization model based on dose–effect relationships [105,106].

#### 5.1.3. Limitations of the Evaluation System

Sensory analysis still relies on traditional subjective scoring methods, focusing primarily on external characteristics such as color, aroma, taste, and texture. This approach is easily influenced by individual preferences and lacks precise analysis of microstructures and nutritional components, such as the retention rate of oxidatively sensitive fatty acids [107,108]. Moreover, studies on the antibacterial mechanisms of alkaline amino acids are often confined to single-strain models in the laboratory, failing to replicate the dynamic responses of complex microbial communities in actual production settings, such as the coexistence of psychrophilic and halophilic bacteria, leading to biased assessments of antibacterial efficacy [109].

### 5.2. Future Outlook

#### 5.2.1. Multi-Scale Mechanism Analysis

By integrating proteomics and molecular dynamics simulations, the specific binding sites and conformational regulation mechanisms of alkaline amino acids with myosin heavy chains are elucidated [22,39,63]. Additionally, macro-transcriptomics techniques are employed to identify the molecular targets of its antibacterial effects (such as changes in membrane permeability [110,111] and inhibition of metabolic pathways) and explore its synergistic effects with natural antibacterial agents (such as ε-polylysine).

However, the high cost and expertise required by omics limit the feasibility of industrial production. For practical quality control, rapid sensor technologies offer industry-ready solutions: electronic tongue systems objectively evaluate sensory profiles (e.g., salty, umami, and bitter tastes) in real time [112], replacing subjective panels and enabling data-driven adjustments [113], while portable NMR quantifies water distribution dynamics to predict shelf life without specialized infrastructure [22]. Complementing these tools, synergistic physical technologies enhance scalability—ultrasound-assisted brining (20 kHz, 300 W) optimizes alkaline amino acid diffusion, reducing salt by 25% while restoring protein extraction efficiency to 75% [24]. These technologies provide a better reference for industrial production.

#### 5.2.2. Cross-Category Standardization Research

In the future, it is crucial to analyze how the biochemical characteristics of different fish species (such as marine vs. freshwater fish and high-fat vs. low-fat fish) influence the function of alkaline amino acids. For instance, high-fat fish, such as mackerel, are rich in ω-3 polyunsaturated fatty acids (PUFA), which may increase their oxidative sensitivity and alter the antioxidant demand threshold [114] for alkaline amino acids like arginine. Freshwater fish, such as tilapia, have looser muscle fibers, which may require adjusting the timing of basic amino acid addition to optimize gel network formation. A database should be established to cover different fish species (such as migratory and bottom-dwelling fish) and processing methods (such as fried and gel products). This database should use response surface methods or machine learning models to determine the optimal addition window based on pH/ionic strength feedback to enhance production controllability. Additionally, for frozen surimi, the synergistic antifreeze mechanism between alkaline amino acids and antifreeze agents (such as trehalose and carboxymethyl cellulose) can be studied [25,56]. The ice crystal inhibition effect can be observed using cryo-SEM.

#### 5.2.3. Establishment of a Quality and Safety Evaluation System

Integrate high-resolution mass spectrometry (HRMS) and atomic force microscopy (AFM) to quantitatively characterize the impact of alkaline amino acids on the oxidation modification sites and pore size of gel networks in surimi. Construct an objective sensory evaluation model using electronic tongue systems to minimize human bias [115,116]. Additionally, conduct dynamic shelf-life experiments to simulate temperature fluctuations in real cold-chain logistics, assessing their antibacterial efficacy.

#### 5.2.4. Technology Integration and Application Expansion

To explore the coupling effect of alkaline amino acids with new processing technologies [117,118,119,120], such as high-pressure homogenization and radio frequency sterilization. HPH (100–400 MPa) can induce the structural unfolding of surimi protein, exposing more binding sites for alkaline amino acids, thereby enhancing its water retention and ion exchange capabilities [7,57]. Additionally, the instantaneous sterilization effect of HPH can complement the antibacterial properties of alkaline amino acids, synergistically improving the texture and safety of low-salt products. Furthermore, Munir S [102] found that the hydroxyl groups in licorice extract can form hydrogen bonds with lysine in surimi protein, enhancing the structural stability and water retention of the protein.

## 6. Conclusions

Alkaline amino acids (arginine, lysine, histidine) enable significant salt reduction (≤0.3 M NaCl) in surimi processing while maintaining gel strength, water retention, and emulsion stability comparable to conventional high-salt systems. Their multi-functionality effectively addresses the key limitations of low-salt formulations. Nevertheless, critical challenges require resolutions, e.g., (1) standardization gaps in optimal dosages across diverse fish species (e.g., high-fat vs. low-fat variants), (2) methodological limitations in evaluating sensory quality and microbiological safety, and (3) regulatory considerations regarding histidine-related allergenicity and inconsistent safety certifications among amino acids. Future research must prioritize establishing species-specific addition protocols and clarifying safety guidelines for industrial implementation. Addressing these priorities will facilitate the adoption of alkaline amino acids as viable salt substitutes, advancing the sustainable production of health-oriented surimi products without compromising quality.

## Data Availability

No new data were created or analyzed in this study. Data sharing is not applicable to this article.

## References

[1] Li W., Wen L., Xiong S., Xiao S., An Y. (2023). Investigation of the effect of chemical composition of surimi and gelling temperature on the odor characteristics of surimi products based on gas chromatography-mass spectrometry/olfactometry. Food Chem..

[2] Sun Q., Zhang Z., Zhang R., Gao R., McClements D.J. (2018). Development of functional or medical foods for oral administration of insulin for diabetes treatment: Gastroprotective edible microgels. J. Agric. Food Chem..

[3] Gao X., You J., Yin T., Xiong S., Liu R. (2023). Simultaneous effect of high intensity ultrasound power, time, and salt contents on gelling properties of silver carp surimi. Food Chem..

[4] Moe S.M., Drüeke T.B. (2003). Management of secondary hyperparathyroidism: The importance and the challenge of controlling parathyroid hormone levels without elevating calcium, phosphorus, and calcium-phosphorus product. Am. J. Nephrol..

[5] Wang X., Luo N., Guo C., Wang X., Xia S. (2024). Enhancing gel strength and saltiness perception of low-salt surimi gels: Synergistic effects of lysine assisted with water bath-microwave heating. Food Biosci..

[6] Wang R., Gao R., Xiao F., Zhou X., Wang H., Xu H., Gong C., Huang P., Zhao Y. (2019). Effect of chicken breast on the physicochemical properties of unwashed sturgeon surimi gels. LWT–Food Sci. Technol..

[7] Rodrigues F.M., Rosenthal A., Tiburski J.H., Cruz A.G.D. (2015). Alternatives to reduce sodium in processed foods and the potential of high pressure technology. Food Sci. Technol..

[8] Xiong Z., Shi T., Zhang W., Kong Y., Yuan L., Gao R. (2021). Improvement of gel properties of low salt surimi using low-dose L-arginine combined with oxidized caffeic acid. LWT–Food Sci. Technol..

[9] Wickline S.A., Hou K.K., Pan H. (2023). Peptide-based nanoparticles for systemic extrahepatic delivery of therapeutic nucleotides. Int. J. Mol. Sci..

[10] Wang X., Li M., Monto A.R., Yuan L., Jin W., Gao R. (2024). Enhancement of the gelling properties of *Aristichthys nobilis*: Insights into intermolecular interactions between okra polysaccharide and myofibrillar protein. Curr. Res. Food Sci..

[11] Monto A.R., Li M., Wang X., Wijaya G.Y.A., Shi T., Xiong Z., Yuan L., Jin W., Li J., Gao R. (2021). Recent developments in maintaining gel properties of surimi products under reduced salt conditions and use of additives. Crit. Rev. Food Sci. Nutr..

[12] Yu C., Hu W., Chen L., Ouyang K., Chen H., Lin S., Wang W. (2025). Basic Amino Acids as Salt Substitutes in Low-Salt Gel-Based Meat Products: A Comprehensive Review of Mechanisms, Benefits, and Future Perspectives. Foods.

[13] Guo X., Gao F., Zhang Y., Peng Z., Jamali M.A. (2021). Effect of l-histidine and l-lysine on the properties of oil-in-water emulsions stabilized by porcine myofibrillar proteins at low/high ionic strength. LWT.

[14] Oh N.S., Lee H.A., Lee J.Y., Joung J.Y., Lee K.B., Kim Y., Lee K.W., Kim S.H. (2013). The dual effects of Maillard reaction and enzymatic hydrolysis on the antioxidant activity of milk proteins. J. Dairy Sci..

[15] Jiang Y.I., Li D., Tu J., Zhong Y., Zhang D., Wang Z., Tao X. (2021). Mechanisms of change in gel water-holding capacity of myofibrillar proteins affected by lipid oxidation: The role of protein unfolding and cross-linking. Food Chem..

[16] Henderson C.A., Gomez C.G., Novak S.M., Mi-Mi L., Gregorio C.C. (2017). Overview of the muscle cytoskeleton. Compr. Physiol..

[17] Tang S., Feng G., Gao R., Ren J., Zhou X., Wang H., Xu H., Zhao Y., Zeng M. (2019). Thermal gel degradation (Modori) in sturgeon (*Acipenseridae*) surimi gels. J. Food Sci..

[18] Pan C., Zhang X., Chen S., Xue Y., Wang Y., Wu Y. (2024). Analysis of quality-related proteins in golden pompano (*Trachinotus ovatus*) fillets with modified atmosphere packaging under superchilling storage. Food Sci. Hum. Wellness.

[19] Zhang L., Li Q., Bao Y., Tan Y., Lametsch R., Hong H., Luo Y. (2022). Recent advances on characterization of protein oxidation in aquatic products: A comprehensive review. Crit. Rev. Food Sci. Nutr..

[20] Shulpekova Y.O., Ostrovskaya A.A., Tumasheva A.A., Deeva T.A., Kopylov A.T., Malsagova K.A., Kaysheva A.L., Ivashkin V.T. (2021). Food Intolerance: The Role of Histamine. Nutrients.

[21] Zhao Y., Wei G., Li J., Tian F., Zheng B., Gao P., Zhou R. (2023). Comparative study on the effect of different salts on surimi gelation and gel properties. Food Hydrocolloids.

[22] Gao R., Wang Y., Mu J., Shi T., Yuan L. (2018). Effect of L-histidine on the heat-induced aggregation of bighead carp (*Aristichthys nobilis*) myosin in low/high ionic strength solution. Food Hydrocolloids.

[23] Bao Y., Yan D., Xu G., Hong H., Gao R. (2024). Effects of chopping temperature on the gel quality of silver carp (*Hypophthalmichthys molitrix*) surimi: Insight from gel-based proteomics. J. Sci. Food Agric..

[24] Zhang L., Li Q., Shi J., Zhu B., Luo Y. (2018). Changes in chemical interactions and gel properties of heat-induced surimi gels from silver carp (*Hypophthalmichthys molitrix*) fillets during setting and heating: Effects of different washing solutions. Food Hydrocolloids.

[25] Zhang C., Chen L., Lu M., Ai C., Cao H., Xiao J., Zhong S., Teng H. (2023). Effect of cellulose on gel properties of heat-induced low-salt surimi gels: Physicochemical characteristics, water distribution and microstructure. Food Chem. X.

[26] Wang Y., Zhao J., Zhang W., Liu C., Jauregi P., Huang M. (2020). Modification of heat-induced whey protein gels by basic amino acids. Food Hydrocolloids.

[27] Zhou C., Hu J., Yu X., Yagoub A.E.A., Zhang Y., Ma H., Gao X., Otu P.N.Y. (2017). Heat and/or ultrasound pretreatments motivated enzymolysis of corn gluten meal: Hydrolysis kinetics and protein structure. LWT–Food Sci. Technol..

[28] Cheng Y., Shi X., Yeboah G.B., Chen L., Wu J. (2024). Effect of multi-mode divergent ultrasound pretreatment on hardness, microstructure and digestion of acid-induced whey protein gels. Foods.

[29] Cheng Y., Donkor P., Ren X., Wu J., Agyemang K., Ayim I., Ma H. (2019). Effect of ultrasound pretreatment with mono-frequency and simultaneous dual frequency on the mechanical properties and microstructure of whey protein emulsion gels. Food Hydrocolloids.

[30] Wu Y.H.S., Lin Y., Wang S., Lin D., Chen J., Chen Y. (2022). Effects of washing step and salt-addition levels on textural and quality properties in the chicken-surimi products. Poult. Sci..

[31] Gao X., Xie Y., Yin T., Hu Y., You J., Xiong S., Liu R. (2021). Effect of high intensity ultrasound on gelation properties of silver carp surimi with different salt contents. Ultrason. Sonochem..

[32] Zhou H., Pang X. (2018). Electrostatic interactions in protein structure, folding, binding, and condensation. Chem. Rev..

[33] Golovanov A.P., Hautbergue G.M., Wilson S.A., Lian L. (2004). A simple method for improving protein solubility and long-term stability. J. Am. Chem. Soc..

[34] Shi H., Zhou T., Wang X., Zou Y., Wang D., Xu W. (2021). Effects of the structure and gel properties of myofibrillar protein on chicken breast quality treated with ultrasound-assisted potassium alginate. Food Chem..

[35] Xue C., Pei Z., Wen P., Chin Y., Hu Y. (2023). Effects of pH and NaCl on the Spatial Structure and Conformation of Myofibrillar Proteins and the Emulsion Gel System—Insights from Computational Molecular Dynamics on Myosin of Golden Pompano. Gels.

[36] Deitiker P.R., Epstein H.F. (1993). Thick filament substructures in *Caenorhabditis elegans*: Evidence for two populations of paramyosin. J. Cell Biol..

[37] Lopez-Enriquez R.L., Ocano-Higuera V.M., Torres-Arreola W., Ezquerra-Brauer J.M., Marquez-Rios E. (2015). Chemical and functional characterization of sarcoplasmic proteins from giant squid (*Dosidicus gigas*) mantle. J. Chem..

[38] Gould J., Wolf B. (2018). Interfacial and emulsifying properties of mealworm protein at the oil/water interface. Food Hydrocolloids.

[39] Wang H., Zhang J., Xu Y., Mi H., Yi S., Gao R., Li X., Li J. (2023). Effects of chickpea protein-stabilized Pickering emulsion on the structure and gelling properties of hairtail fish myosin gel. Food Chem..

[40] Wang W., Ma S., Shao Q., Yi S. (2024). Effects of Soy Protein Isolate and Inulin Conjugate on Gel Properties and Molecular Conformation of Spanish Mackerel Myofibrillar Protein. Foods.

[41] Depta P.N., Gurikov P., Schroeter B., Forgács A., Kalmár J., Paul G., Marchese L., Heinrich S., Dosta M. (2021). DEM-based approach for the modeling of gelation and its application to alginate. J. Chem. Inf. Model..

[42] Barcenilla C., Álvarez-Ordóñez A., López M., Alvseike O., Prieto M. (2022). Microbiological safety and shelf-life of low-salt meat products—A Review. Foods.

[43] Zhang Y., Li J., Teng S., Peng Z., Jamali M.A. (2023). Quality improvement of prerigor salted ground chicken breast with basic amino acids at low NaCl level. Poult. Sci..

[44] Wu D., Wang H., Guo X., Zhang Z., Gao Z., Gao S., Liu Z., Rao S., Meng X. (2023). Insight into the mechanism of enhancing myofibrillar protein gel hardness by ultrasonic treatment combined with insoluble dietary fiber from oat. LWT–Food Sci. Technol..

[45] Calligaris S., Plazzotta S., Valoppi F., Anese M. (2018). Combined high-power ultrasound and high-pressure homogenization nanoemulsification: The effect of energy density, oil content and emulsifier type and content. Food Res. Int..

[46] Wang Y., Yan J., Ding Y., Rashid M.T., Ma H. (2021). Effect of sweep frequency ultrasound and fixed frequency ultrasound thawing on gelling properties of myofibrillar protein from quick-frozen small yellow croaker and its possible mechanisms. LWT–Food Sci. Technol..

[47] Hand L.W., Terrell R.N., Smith G.C. (1982). Effects of complete or partial replacement of sodium chloride on processing and sensory properties of hams. J. Food Sci..

[48] Fuentes A., Fernández-Segovia I., Serra J.A., Barat J.M. (2011). Influence of sodium replacement and packaging on quality and shelf life of smoked sea bass (*Dicentrarchus labrax* L.). LWT-Food Sci. Technol..

[49] Kim T.K., Yong H.I., Jung S., Kim H.M., Choi Y.S. (2021). Effect of reducing sodium chloride based on the sensory properties of meat products and the improvement strategies employed: A review. J. Anim. Sci. Technol..

[50] Fullenkamp D.E., He L., Barrett D.G., Burghardt W.R., Messersimth P.B. (2013). Mussel-inspired histidine-based transient network metal coordination hydrogels. Macromolecules.

[51] Nooshkam M., Varidi M., Zareie Z., Alkobeisi F. (2023). Behavior of protein-polysaccharide conjugate-stabilized food emulsions under various destabilization conditions. Food Chem. X.

[52] Wang Y., Hu X., Han J., Ni L., X Tang X., Hu Y., Cen T. (2016). Integrated method of thermosensitive triblock copolymer–salt aqueous two-phase extraction and dialysis membrane separation for purification of *Lycium barbarum* polysaccharide. Food Chem..

[53] Gouseti O., Larsen M.E., Amin A., Bakalis S., Petersen I.L., Lametsch R., Jensen P.E. (2023). Applications of enzyme technology to enhance transition to plant proteins: A review. Foods.

[54] Leng W., Li Y., Liang X., Yuan L., Li X., Gao R. (2025). Thermo-reversible gelation and enhanced umami perception of myofibrillar proteins induced by protein-glutaminase-mediated deamidation. Food Chem..

[55] Lin J., Meng H., Guo X., Tang Z., Yu S. (2023). Natural aldehyde-chitosan Schiff base: Fabrication, pH-responsive properties, and vegetable preservation. Foods.

[56] Javed M., Huang H., Ma Y., Ettoumi F.E., Wang L., Xu Y., EI-Seedi H.R., Ru Q., Luo Z. (2023). Construction of self-assembled nanocellulose crystals/chitosan nanobubbles composite hydrogel with improved gallic acid release property. Food Chem..

[57] Rode T.M., Rotabakk B.T. (2021). Extending shelf life of desalted cod by high pressure processing. Innov. Food Sci. Emerg. Technol..

[58] Pan J., Zhang Z., Mintah B.K., Xu H., Dabbour M., Cheng Y., Dai C., He R., Ma H. (2022). Effects of nonthermal physical processing technologies on functional, structural properties and digestibility of food protein: A review. J. Food Process Eng..

[59] Shi T., Yuan L., Mu J., Gao R. (2019). The effect of Arginine, Lysine and Histidine in the myosin secondary structure by circular dichroism and Raman spectroscopy. CyTA-J. Food.

[60] Gao R., Wang X., Shi T., Wijaya G.Y.A., Bai F., Wang J., Jin W., Yuan L. (2021). Enhanced physical properties of reduced-salt surimi gels from Amur sturgeon (*Acipenser schrenckii*) by L-arginine and L-histidine. J. Food Process. Preserv..

[61] Wang Y., Mei Y., Du R., Zhang S., Wang Q., Dao X., Li N., Wang L., Wang L., He R. (2024). Arginine as a regulator of antioxidant and gel formation in yak Myofibrillar proteins: Efficacy and mechanistic insights. Food Chem. X.

[62] Wang X., Feng T., Wang X., Zhang X., Xia S. (2021). Gelation and microstructural properties of fish myofibrillar protein gels with the incorporation of L-lysine and L-arginine at low ionic strength. J. Sci. Food Agric..

[63] Shi T., Xiong Z., Jin W., Yuan L., Sun Q., Zhang Y., Li X., Gao R. (2020). Suppression mechanism of L-arginine in the heat-induced aggregation of bighead carp (*Aristichthys nobilis*) myosin: The significance of ionic linkage effects and hydrogen bond effects. Food Hydrocolloids.

[64] Zhang Y., Shao F., Wan X., Zhang H., Cai M., Hu K., Duan Y. (2023). Effects of rapeseed protein addition on soybean protein-based textured protein produced by low-moisture extrusion: Changes in physicochemical attributes, structural properties and barrel flow behaviors. Food Hydrocolloids.

[65] Mason P.E., Neilson G.W., Dempsey C.E., Barnes A.C., Cruickshank J.M. (2003). The hydration structure of guanidinium and thiocyanate ions: Implications for protein stability in aqueous solution. Proc. Natl. Acad. Sci. USA.

[66] Shi T., Wang X., Li M., Xiong Z., McClements D.J., Bao Y., Song T., Li J., Jin W., Gao R. (2022). Mechanism of low-salt surimi gelation induced by microwave heating combined with l-arginine and transglutaminase: On the basis of molecular docking between l-arginine and myosin heavy chain. Food Chem..

[67] Lv G., Wang H., Wei X., Liu X., Yang W., Aalim H., Capanoglu E., Zou X., Battion M., Zhang D. (2023). Cooking-induced oxidation and structural changes in chicken protein: Their impact on *in vitro* gastrointestinal digestion and intestinal flora fermentation characteristics. Foods.

[68] Schönichen A., Webb B.A., Jacobson M.P., Barber D.L. (2013). Considering protonation as a posttranslational modification regulating protein structure and function. Annu. Rev. Biophys..

[69] Trujillo C., Rodriguez-Sanz A.A., Rozas I. (2015). Aromatic Amino Acids–Guanidinium Complexes through Cation–π Interactions. Molecules.

[70] Park S., Lee Y., Jho Y., Hwang D.S. (2023). Molecular hydration tunes the cation–π interaction strength in aqueous solution. Adv. Mater. Interfaces.

[71] Sun W., Xue B., Fan Q., Tao R., Wang C., Wang X., Li Y., Qin M., Wang W., Chen B. (2020). Molecular engineering of metal coordination interactions for strong, tough, and fast-recovery hydrogels. Sci. Adv..

[72] Zhang Q., Ames J.M., Smith R.D., Baynes J.W., Metz T.O. (2009). A perspective on the Maillard reaction and the analysis of protein glycation by mass spectrometry: Probing the pathogenesis of chronic disease. J. Proteome Res..

[73] Koniev O., Wagner A. (2015). Developments and recent advancements in the field of endogenous amino acid selective bond forming reactions for bioconjugation. Chem. Soc. Rev..

[74] Campbell K.S. (2014). Dynamic coupling of regulated binding sites and cycling myosin heads in striated muscle. J. Gen. Physiol..

[75] Li X., Wang W., Wang S., Shen Y., Pan J., Dong X., Li S. (2022). The solubility and structures of porcine myofibrillar proteins under low-salt processing conditions as affected by the presence of L-lysine. Foods.

[76] Jiang C., Wang X., Hou B., Hao C., Li X., Wu J. (2020). Construction of a lignosulfonate-lysine hydrogel for the adsorption of heavy metal ions. J. Agric. Food Chem..

[77] Yan D., Xu W., Yu Q., You J., Gao R., Bao Y. (2024). Pre-rigor salting improves gel strength and water-holding of surimi gel made from snakehead fish (*Channa argus*): The role of protein oxidation. Food Chem..

[78] Liu Y., Zhang L., Gao S., Bao Y., Tan Y., Luo Y., Li X., Hong H. (2021). Effect of protein oxidation in meat and exudates on the water holding capacity in bighead carp (*Hypophthalmichthys nobilis*) subjected to frozen storage. Food Chem..

[79] Zhang J., Wen C., Duan Y., Zhang H., Ma H. (2021). Structure and functional properties of watermelon seed protein–glucose conjugates prepared by different methods. LWT–Food Sci. Technol..

[80] Guo X., Peng Z., Zhang Y., Liu B., Cui Y. (2015). The solubility and conformational characteristics of porcine myosin as affected by the presence of L-lysine and L-histidine. Food Chem..

[81] Chen X., Li Y., Zhou R., Liu Z., Lu F., Lin H., Xu X., Zhou G. (2016). L-histidine improves water retention of heat-induced gel of chicken breast myofibrillar proteins in low ionic strength solution. Int. J. Food Sci. Technol..

[82] Nakashige T.G., Stephan J.R., Cunden L.S., Brophy M.B., Wommack A.J., Keegan B.C., Shearer J.M., Nolan E.M. (2016). The hexahistidine motif of host-defense protein human calprotectin contributes to zinc withholding and its functional versatility. J. Am. Chem. Soc..

[83] Mao C., Wu J., Zhang X., Ma F., Cheng Y. (2020). Improving the solubility and digestibility of potato protein with an online ultrasound-assisted pH shifting treatment at medium temperature. Foods.

[84] Yu C., Chen L., Ouyang K., Chen H., Lin S., Wang W. (2025). Effect of the Partial Substitution of NaCl with L-Arg on the Gel Properties and Aggregation Behavior of Beef Myosin. Foods.

[85] Lin J., Sun P., Zhao Y., Du X., Ren X., Man H., Li D. (2024). Effect of L-lysine on heat-induced aggregation behavior of Antarctic krill (*Euphausia superba*) Myofibrillar Protein. Food Bioprocess Technol..

[86] Berhanu W.M., Masunov A.E. (2014). Full length amylin oligomer aggregation: Insights from molecular dynamics simulations and implications for design of aggregation inhibitors. J. Biomol. Struct. Dyn..

[87] Huang L., Ding X., Li Y., Ma H. (2019). The aggregation, structures and emulsifying properties of soybean protein isolate induced by ultrasound and acid. Food Chem..

[88] Scarff C.A., Carrington G., Casas-Mao D., Chalovich J.M., Knight P.J., Ranson N.A., Peckham M. (2020). Structure of the shutdown state of myosin-2. Nature.

[89] Mu Y., Sun J., Obadi M., Chen Z., Xu B. (2020). Effects of saccharides on the rheological and gelling properties and water mobility of egg white protein. Food Hydrocolloids.

[90] Zhang Z., Yang T., Wang Y., Liu J., Shi W., Hu H., Meng Y., Meng X., He R. (2023). Influence of multi-frequency ultrasound treatment on conformational characteristics of beef myofibrillar proteins with different degrees of doneness. Foods.

[91] Li L., Han L., Fu Q., Li Y., Liang Z., Su J., Li B. (2012). Formation and inhibition of ε-(carboxymethyl) lysine in saccharide-lysine model systems during microwave heating. Molecules.

[92] Mao C., Wu J., Cheng Y., Chen T., Ren X., Ma H. (2021). Physicochemical properties and digestive kinetics of whey protein gels filled with potato and whey protein mixture emulsified oil droplets: Effect of protein ratios. Food Funct..

[93] Pan J., Li C., Liu X., He L., Zhang M., Hang S., Huang S., Liu Y., Zhang Y., Jin J. (2022). A multivariate insight into the organoleptic properties of porcine muscle by ultrasound-assisted brining: Protein oxidation, water state and microstructure. LWT–Food Sci. Technol..

[94] Li S., Li M., Cao H., Guan X., Zhang Y., Huang K., Zhang Y. (2022). The intervening effect of L-lysine on the gel properties of wheat gluten under microwave irradiation. Food Chem. X.

[95] Henao A., Ruiz G.N., Steinke N., Cerveny S., Macovez R., Guàrdia E., Busch S., McLain S.E., Lorenz C.D., Pardo L.C. (2020). On the microscopic origin of the cryoprotective effect in lysine solutions. Phys. Chem. Chem. Phys..

[96] Hao D., Tu X., Zhang X., Guo S., Sun L., Li J., Wang D., Xu W., Li P. (2024). Effects of protease inactivation on textural quality of yellow-feathered chicken meat and the possible mechanism based on myofibrillar protein. Food Control.

[97] Jin J., Ma H., Wang W., Luo M., Wang B., Qu W., He R., Owusu J., Li Y. (2016). Effects and mechanism of ultrasound pretreatment on rapeseed protein enzymolysis. J. Sci. Food Agric..

[98] Huang L., Ding X., Dai C., Ma H. (2017). Changes in the structure and dissociation of soybean protein isolate induced by ultrasound-assisted acid pretreatment. Food Chem..

[99] Li L., Cai R., Wang P., Xu X., Zhou G., Sun J. (2018). Manipulating interfacial behavior and emulsifying properties of myosin through alkali-heat treatment. Food Hydrocolloids.

[100] Man H., Sun P., Lin J., Ren X., Li D. (2024). Based on hydrogen and disulfide-mediated bonds, L-lysine and L-arginine enhanced the gel properties of low-salt mixed shrimp surimi (*Antarctic krill* and Pacific White Shrimp). Food Chem..

[101] Chen X., Zou Y., Han M., Pan L., Xing T., Xu X., Zhou G. (2016). Solubilization of myosin in a solution of low ionic strength l-histidine: Significance of the imidazole ring. Food Chem..

[102] Munir S., Javed M., Hu Y., Liu Y., Xiong S. (2020). The effect of acidic and alkaline pH on the physico-mechanical properties of surimi-based edible films incorporated with green tea extract. Polymers.

[103] Xiong Z., Shi T., Jin W., Bao Y., Monto A.R., Yuan L., Gao R. (2022). Gel performance of surimi induced by various thermal technologies: A review. Crit. Rev. Food Sci. Nutr..

[104] Xiong Z., Wang X., Li M., Shi T., Jin W., Li J., Yuan L., Gao R. (2023). Investigation of the enhancement mechanism of ethanol addition on the gel performance of heat-induced surimi. J. Food Eng..

[105] Hou F., Ding W., Qu W., Oladejo A.O., Xiong F., Zhang W., He R., Ma H. (2017). Alkali solution extraction of rice residue protein isolates: Influence of alkali concentration on protein functional, structural properties and lysinoalanine formation. Food Chem..

[106] Qu W., Zhang X., Han X., Wang Z., He R., Ma H. (2018). Structure and functional characteristics of rapeseed protein isolate–dextran conjugates. Food Hydrocolloids.

[107] Karabulut G., Subasi B.G., Ivanova P., Goksen G., Chalova V., Capanoglu E. (2025). Towards sustainable and nutritional-based plant protein sources: A review on the role of rapeseed. Food Res. Int..

[108] Feng R., Yu Q., Bao Y., Chen L., Luo Y., Tan Y., Hong H. (2024). Myofibrillar protein lipoxidation in fish induced by linoleic acid and 4-hydroxy-2-nonenal: Insights from LC-MS/MS analysis. Food Res. Int..

[109] Wang L., Wang X., Bai F., Fang Y., Wang J., Gao R. (2019). The anti-skin-aging effect of oral administration of gelatin from the swim bladder of Amur sturgeon (*Acipenser schrenckii*). Food Funct..

[110] Sun J., Mu Y., Mohammed O., Dong S., Xu B. (2020). Effects of single-mode microwave heating and dextran conjugation on the structure and functionality of ovalbumin–dextran conjugates. Food Res. Int..

[111] Molchanova N., Hansen P.R., Franzyk H. (2017). Advances in development of antimicrobial peptidomimetics as potential drugs. Molecules.

[112] Wang X.J. (2021). The Enhancement of Saltiness Perception in Fish Products by Microwave Treatment and the Processing Adaptability of Reduced-Salt Surimi. Ph.D. Thesis.

[113] Campagnol P.C.B., dos Santos B.A., Morgano M.A., Terra N.N., Pollonio M.A.R. (2011). Application of lysine, taurine, disodium inosinate and disodium guanylate in fermented cooked sausages with 50% replacement of NaCl by KCl. Meat Sci..

[114] Zhu Y., Li C., Cui H., Lin L. (2020). Plasma enhanced-nutmeg essential oil solid liposome treatment on the gelling and storage properties of pork meat batters. J. Food Eng..

[115] Zhai Y., Luan A., Yang Z., Rong Z., Liu Y., Wang F., Li X. (2024). The impacts of cold plasma on the taste and odor formation of dried silver carp products. Food Chem..

[116] Matricardi P., Cencetti C., Ria R., Alhaique F., Coviello T. (2009). Preparation and characterization of novel gellan gum hydrogels suitable for modified drug release. Molecules.

[117] Yang J., Duan Y., Zhang H., Huang F., Wan C., Cheng C., Wang L., Peng D., Deng Q. (2023). Ultrasound coupled with weak alkali cycling-induced exchange of free sulfhydryl–disulfide bond for remodeling interfacial flexibility of flaxseed protein isolates. Food Hydrocolloids.

[118] Wen C., Zhang J., Duan Y., Zhang H., Ma H. (2019). A mini-review on brewer’s spent grain protein: Isolation, physicochemical properties, application of protein, and functional properties of hydrolysates. J. Food Sci..

[119] Li Y., Zhang Z., Ren W., Wang Y., Mintah B.K., Dabbour M., Hou Y., He R., Cheng Y., Ma H. (2021). Inhibition effect of ultrasound on the formation of lysinoalanine in rapeseed protein isolates during pH shift treatment. J. Agric. Food Chem..

[120] Sun J., Huang Y., Liu T., Jing H., Zhang F., Obadi M., Xu B. (2022). Evaluation of crosslinking sites of egg white protein–polyphenol conjugates: Fabricated using a conventional and ultrasound-assisted free radical technique. Food Chem..

